# Building an Extracorporeal Membrane Oxygenation Digital Twin Using High-Resolution Patient Data: An Artificial Intelligence Model for Virtual Reality Simulation

**DOI:** 10.1093/icvts/ivag047

**Published:** 2026-02-19

**Authors:** Samuel Max, Mounir Bourass, Andre van der Mee Mendes, Daniel van der Mee Mendes, Bram Schalkwijk, Zaheer Babar, Lydia Lim, Carlos Elzo Kraemer, Brian Kaufman, Antony van Dijk, Renske Brekelmans, Priscilla Westbroek, Abdelrhman Mostafa Ali, Juan Hugo, Robert Klautz, Jerry Braun, Edris Mahtab

**Affiliations:** Department of Cardiothoracic Surgery, Leiden University Medical Center, Leiden, 2333 ZA, The Netherlands; Department of Cardiothoracic Surgery, Leiden University Medical Center, Leiden, 2333 ZA, The Netherlands; Department of Cardiothoracic Surgery, Leiden University Medical Center, Leiden, 2333 ZA, The Netherlands; Department of Cardiothoracic Surgery, Leiden University Medical Center, Leiden, 2333 ZA, The Netherlands; Department of Cardiothoracic Surgery, Erasmus MC University Medical Center, Rotterdam, 3015 GD, The Netherlands; Department of Cardiothoracic Surgery, Leiden University Medical Center, Leiden, 2333 ZA, The Netherlands; Department of Cardiothoracic Surgery, Erasmus MC University Medical Center, Rotterdam, 3015 GD, The Netherlands; Department of Cardiothoracic Surgery, Leiden University Medical Center, Leiden, 2333 ZA, The Netherlands; Department of Cardiothoracic Surgery, Leiden University Medical Center, Leiden, 2333 ZA, The Netherlands; Department of Medicine, NYU Langone Health, New York, NY 10010, United States; Department of Anesthesiology, NYU Langone Health, New York, NY 10010, United States; Department of Neurosurgery, NYU Langone Health, New York, NY 10010, United States; Department of Cardiothoracic Surgery, Erasmus MC University Medical Center, Rotterdam, 3015 GD, The Netherlands; Department of Cardiothoracic Surgery, Erasmus MC University Medical Center, Rotterdam, 3015 GD, The Netherlands; Department of Cardiothoracic Surgery, Erasmus MC University Medical Center, Rotterdam, 3015 GD, The Netherlands; Department of Cardiothoracic Surgery, Leiden University Medical Center, Leiden, 2333 ZA, The Netherlands; Department of Cardiothoracic Surgery, Leiden University Medical Center, Leiden, 2333 ZA, The Netherlands; Department of Cardiothoracic Surgery, Leiden University Medical Center, Leiden, 2333 ZA, The Netherlands; Department of Cardiothoracic Surgery, Amsterdam UMC, Amsterdam, 1105 AZ, The Netherlands; Department of Cardiothoracic Surgery, Leiden University Medical Center, Leiden, 2333 ZA, The Netherlands; Department of Cardiothoracic Surgery, Amsterdam UMC, Amsterdam, 1105 AZ, The Netherlands; Department of Cardiothoracic Surgery, Leiden University Medical Center, Leiden, 2333 ZA, The Netherlands

**Keywords:** digital twin, extracorporeal membrane oxygenator, training, neural network, artificial intelligence, virtual reality

## Abstract

**Objectives:**

Extracorporeal membrane oxygenation (ECMO) is a life-saving therapy for severe cardiopulmonary failure, but structured training remains constrained by costs, logistics, and the absence of validated high-fidelity simulators. This study aimed to develop an ECMO digital twin capable of supporting training in virtual reality (VR).

**Methods:**

We integrated high-frequency ECMO machine data with electronic health record information from 335 patients across 2 centres. Data streams were synchronized at a 30-s resolution. A hierarchical 2-stage system was designed: Model 1 predicted ECMO device outputs, while Model 2 combined those outputs with patient vital functions such as heart rate and blood pressure. This model was integrated into a VR simulation and underwent testing by 21 experts.

**Results:**

Model 2 demonstrated root mean square errors (RMSEs) of 15.23 mm Hg (diastolic arterial blood pressure), 19.50 BPM (heart rate), 2.94% (peripheral oxygen saturation), and 1.42 mm Hg (end-tidal carbon dioxide) on the test set. Neural networks produced clinically coherent predictions. The models were implemented in an unreal engine-based VR simulator using the open neural network exchange format, with real-time latency inference and scenario switching. Expert testing confirmed good performance and clinically plausible physiological responses in the simulation.

**Conclusions:**

High-resolution ECMO data can be transformed into a digital twin for VR training. This framework broadens access to advanced ECMO education and establishes a foundation for multicentre validation, federated learning, and future expansion towards a critical-care digital-twin platform.

## Graphical abstract

**Figure:**
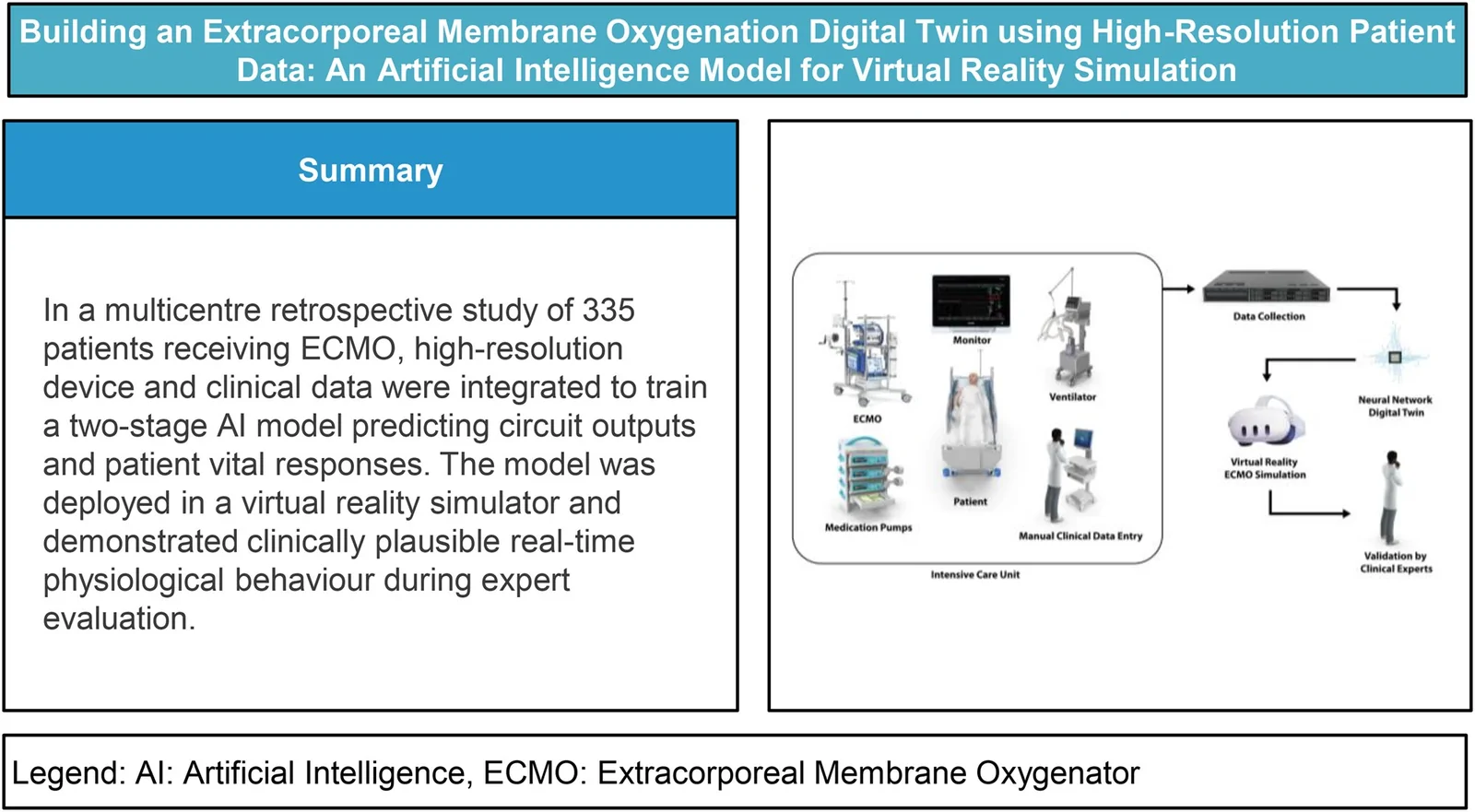

## INTRODUCTION

Extracorporeal membrane oxygenation (ECMO) is a critical intervention in the management of severe cardiopulmonary failure, offering a bridge to recovery or transplantation for patients who are otherwise refractory to conventional therapies. ECMO is a complex therapy, and inadequate knowledge or delayed decision-making can result in morbidity and mortality.^1^ Comprehensive training for healthcare professionals is therefore imperative. This training is labour intensive and expensive. It requires a dedicated physical simulation unit and a training ECMO machine, which limits access and results in a low percentage of ECMO centres running a formal training programme.^2^ Further to this, according to Duinmeijer and colleagues, no high-fidelity ECMO simulators exist that satisfy all of the required validity criteria.^3^

Digital ECMO simulations, and particularly virtual reality (VR) are promising modalities to ameliorate this gap, and improve access to high-fidelity simulations. Our VR simulation platform for cardiothoracic surgery has been shown to be realistic, useful, and easy to use in the context of educating perfusionists, surgical, residents and allied healthcare professionals.^4–6^ The qualitative data from these studies demonstrate that to achieve true high-fidelity simulation, a high-fidelity simulation of the ECMO-patient mechano-physiological interaction is required.

Two broad approaches have historically been used for generating such physiological responses, including mechanistic models range from 0D lumped-parameter cardiovascular models to 1D haemodynamic simulators with spatial resolution.^7–9^ These models are attractive for their computational speed. However, most published ECMO variants lack full clinical validation and can produce widely different outputs for a given RPM. In addition, they are often unsuitable for simulating patients in extremis and may be computationally demanding.^10–13^ Only a minority of models are clinically validated, and few are coupled to haemodynamics for beat-to-beat changes.^14–16^ Off-the-shelf engines such as Pulse Physiology Engine (Kitware, Inc., Clifton Park, New York, United States) offer well validated physiology simulations but currently do not support ECMO out of the box and are too computationally demanding when adequately parameterised.^17^

An as-of-yet unexplored path is modelling that learns the mechano-physiological interactions directly from high-resolution ECMO data and corresponding clinical data. This approach is derived from the concept of a digital twin. It promises to enable patient-specific simulation across a wide variety of clinical scenarios and to adapt dynamically to user inputs, even in physiologically extreme settings.^18^^,^^19^ Machine learning techniques, particularly neural network-based architectures, are well-suited to modelling such complex, non-linear interactions.

In this study, we present a methodology developed to integrate ECMO-derived device data with detailed patient-level clinical information, and to construct a 2-stage predictive model. Additionally, we demonstrate that this model can be integrated in a VR simulation environment with modest computational impact, and assessed the model outputs for clinical coherence.

## METHODS

### Study design

Ethical approval was obtained from the Erasmus MC Medical Ethics Committee (MEC-2023-0262; March 24, 2023) and the Leiden University Medical Center (DAP/tak/0932025; May 7, 2025). Data were collected both retrospectively and prospectively for patients in both aforementioned centres undergoing ECMO therapy between January 2020 and July 2025 and were stored in institutional research databases for ongoing and future scientific use. The establishment, storage, and secondary use of these data were reviewed and approved by the respective research ethics committees and were conducted in accordance with the principles of the World Medical Association Declaration of Taipei. Owing to the high proportion of patients unable to consent (morbidity/mortality) and the undue burden during acute care, the informed consent requirement was waived by the institutional review boards.

The estimated sample size required was calculated for each category, veno-arterial (VA), and veno-venous (VV) ECMO. With a median ECMO duration of 4 days, and the average interval of 5 min for ECMO data registration, each mode requires an estimated 172 800 time points with 19 000 effective samples after autocorrelation correction. A final sample size of 61 VA and 61 VV patients was calculated. The calculated sample size represented a minimum estimate based on conventional assumptions. During model development, additional patients were included to improve coverage of rare disaster scenarios and improve model performance.

### Data collection and integration

#### Primary data acquisition.

Both centres primarily used the Cardiohelp ECMO platform (Getinge AB, Gothenburg, Sweden). Device logs recorded at 5-minute intervals were exported via the onboard USB after ECMO completion or during machine exchange. These timestamped logs (raw and computed parameters) were linked to clinical records from the electronic health record (EHR).

Continuous patient monitoring data were obtained via the Philips Data Warehouse Connect pipeline (Philips, Eindhoven, the Netherlands) as described by Malunjkar et al.^20^ Waveforms sampled at 25 Hz were downsampled to 30-s intervals to align with ECMO data, which were interpolated to the same 30-s resolution.

Integration of these separate data streams required manual matching based on timestamps and cross-referencing with the perfusion registry. The manual internal clocks in the ECMO devices resulted in temporal drift of the timestamps. This was mitigated by extracting low-frequency ECMO data recorded in the EHR, which facilitated matching with the higher-resolution data sources.

A set of standardized preprocessing procedures was implemented to optimize data quality and temporal alignment. The synchronization of data streams from multiple sources was validated using identifiable reference events, such as the initiation or cessation of ECMO support. In addition to device and standard vital parameters, our database incorporated demographic baseline characteristics of the patient, including their height, weight, sex, medication administration records, and laboratory results, where available, with manual validation for perfusion registry variables.

#### Transition to an automated, high-resolution data pipeline.

To increase the resolution of the data, reduce missed exports and misalignment, we implemented an automated high-frequency pipeline. Bedside devices (patient monitor, ventilator, and infusion pumps) transmitted data to both the EHR at predefined intervals and a continuous data stream to a research server. The overall architecture of the data acquisition system is shown in **Figure 1**.

**Figure 1.**
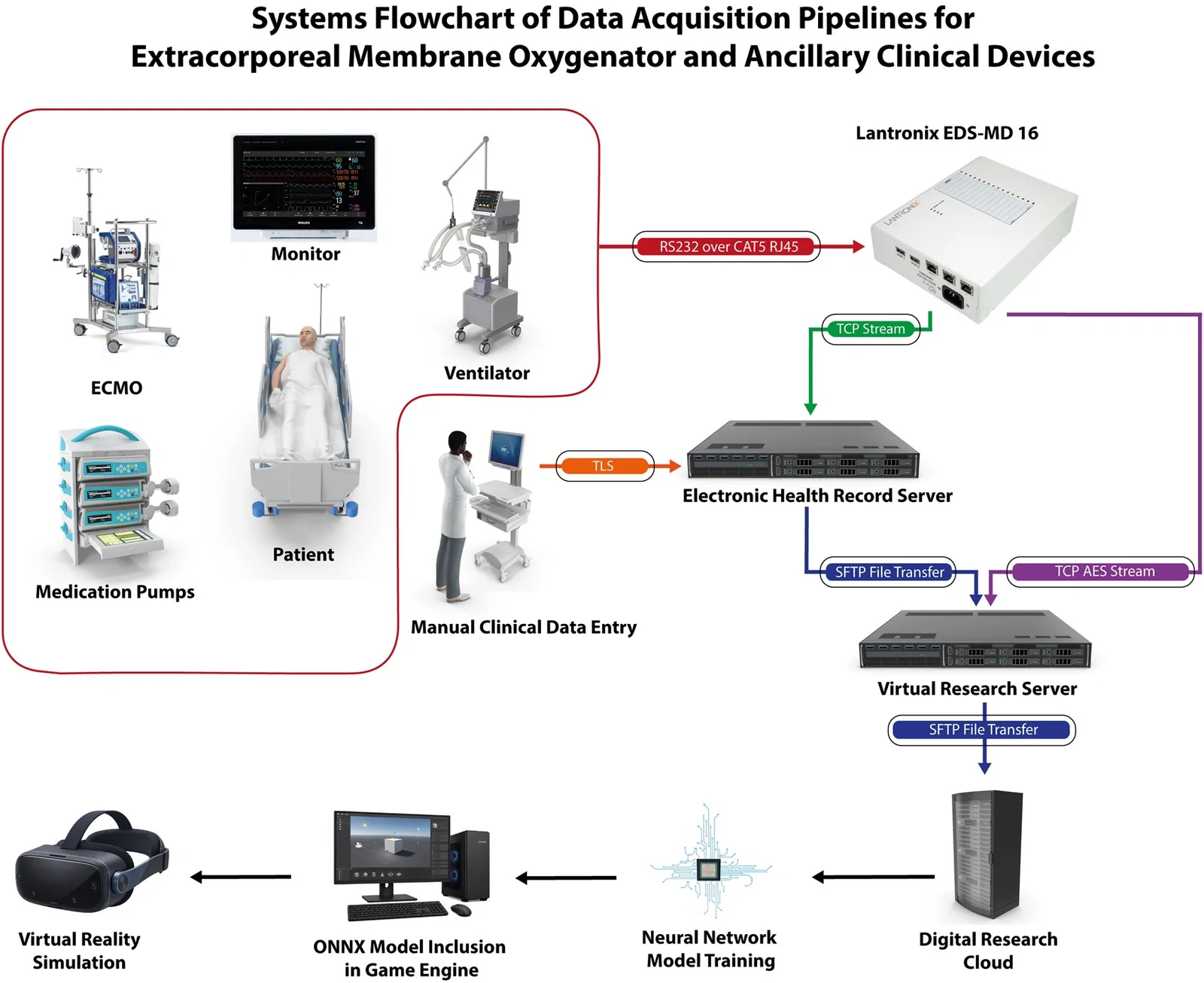
Systems Flowchart of Data Acquisition Pipelines for the Extracorporeal Membrane Oxygenator and Ancillary Clinical Devices. Image credits: ECMO photograph reproduced with permission from Getinge Netherlands B.V. © Getinge, all rights reserved. Permission for re-use should be sought from the rights-holder. Lantronix EDS-MD 16 photograph reproduced with permission from Lantronix. © Lantronix, all rights reserved. Permission for re-use should be sought from the rights-holder. Stock illustrations reproduced under licence from Envato Elements © PixelSquid360. All rights reserved. Permission for re-use should be sought from the rights-holder

### Data preprocessing and feature engineering

The number of patients identified for inclusion and the number of patients matched for the final dataset can be found in **Table 1**. High-frequency variables (eg, heart rate, mean arterial pressure, RPM) were resampled to 30 s. Low-frequency variables (laboratory values, medication administrations) were forward-filled or aggregated to align with the time-series inputs.

**Table 1.** Baseline Demographic and Clinical Characteristics of the Study Population

Characteristic	*N* (%)
Total patients	335 (100%)
Sex	*n* (%)
Female	113 (33.7%)
Male	222 (66.3%)
Age distribution in years	*n* (%)
10-19	11 (3.3%)
20-29	19 (5.7%)
30-39	34 (10.1%)
40-49	72 (21.5%)
50-59	70 (20.9%)
60-69	85 (25.4%)
70-79	36 (10.7%)
80-89	1 (0.3%)
ECMO configuration	*n* (%)
Veno-arterial (VA)	267 (79.7%)
Veno-venous (VV)	62 (18.5%)
Veno-veno-arterial (VVA)	1 (0.3%)
Data availability	*n* (%)
ECMO device data	335 (100%)
Electronic health record data	335 (100%)
Cannula size documented	335 (100%)
High-frequency bedside monitoring data	113 (33.7%)

**Table 2.** Input and Output Variables for the 2 Neural Network Models

	Model 1	Model 2
Input	RPM, cannula size (arterial, venous)	RPM, flow, arterial pressure, venous pressure, inlet pressure
Output	Flow, arterial pressure, venous pressure, inlet pressure	Heart rate, diastolic blood pressure, oxygen saturation, respiratory rate, and end-tidal CO_2_

**Table 3.** Neural Network Architecture for Model 1

Layer	Output shape	Number of parameters
Input	(3)	(0)
ReLu		
Dense (8)	(8)	(32)
ReLu		
Dense (16)	(16)	(144)
ReLu		
Dense (8)	(8)	(136)
Dense (4)	(4)	(36)
Total parameters		(348)

Feature inclusion was guided by domain expertise, literature, and iterative experiments. The final inputs included ECMO device settings, measured ECMO outputs (flow; arterial/venous/inlet pressures), cannula sizes, and patient clinical variables (**Table 2**).

In the collected ECMO dataset, only a limited number of measurements were available at low RPM values. Up sampling of these datapoints led to dense clusters that did not represent the data accurately. We therefore generated sparse synthetic samples spanning 0-300 RPM with plausible ranges for associated outputs and added noise, distributing samples across pseudo-patients to avoid patient-level leakage. This yielded a more uniform operating-range representation and stabilized training at extremes.

### Predictive modelling strategy

#### Two-stage modelling approach.

To capture ECMO machine-patient coupling, we implemented a hierarchical pipeline. Model 1 predicts ECMO mechanical outputs (flow, pressures) from device settings (RPM, sweep gas flow) and cannula sizes. Its predictions, together with original settings and selected patient features, form the input to Model 2, which predicts patient vital signs responses (eg, blood pressure). At inference, Model 1 runs first and streams outputs to Model 2, preserving modularity and interpretability. A detailed overview of the data integration, preprocessing, and validation pipeline is provided in **Supplementary Materials**.

#### Model architecture and training.

Model development followed an iterative strategy. Initial benchmarks were established using linear regression models, followed by more flexible random forest regressors, and culminating in feedforward neural networks with 3 hidden layers, 8-16 nodes, 3 RELU activation functions (**Table 3**). For selected time-dependent prediction tasks, recurrent architectures such as recurrent neural networks and long-short term memory (LSTM) neural networks were also explored, using sliding input windows of 30 s and targets defined at clinically relevant prediction horizons (eg, *t* + 1, *t* + 5). All models were implemented in Python using the TensorFlow library (version [2.19]). Full model hyperparameters, training procedures, and cross-validation details are provided in **Supplementary Materials S3 and S4**.

#### Correction for data imbalance.

A methodological challenge was the imbalance in the distribution of RPM values. Most data points clustered around standard operational settings, while extreme values which while clinically important, occurred less frequently. To mitigate the imbalance in RPM values, the RPM range was discretized into bins, and the training data were resampled to achieve approximately equal numbers of data points per bin. This procedure aimed to ensure adequate representation of both low and high RPM scenarios during model training.

#### Train-rest splitting and cross-validation.

To ensure generalizability and prevent data leakage, the dataset was split at the patient level: all data from a given patient was assigned exclusively to either the training or test set. Given the marked disparity in the number of data points contributed by each patient, the split was stratified to maintain an approximate 70/30 ratio in total data points between training and testing, rather than by patient count. No significant differences were found between the training and test set in terms of baseline demographic characteristics. Model performance was quantified using root mean squared error (RMSE), coefficient of determination (*R*^2^), and, where appropriate, additional metrics such as mean absolute error (MAE).

### Model export and deployment in virtual reality

For deployment in a resource-constrained VR simulation environment, final trained models were exported to the open neural network exchange (ONNX) format, which itself imposed constraints regarding model architecture. Required compute resources strongly impacted the export and optimization process. Export included conversion of model weights and dynamic input axes, together with pre- and post-processing routines to facilitate real-time inference with latencies consistently below 100 ms.

Within the VR application the different models can be preloaded, enabling rapid switching between different clinical scenarios. To conform with ONNX limitations, model architectures were optimized for memory footprint and computational efficiency, while preserving predictive performance. This was achieved by integrating the ONNX Runtime library, enabling on-device running of the AI model on the android-based VR headset. This avoided reliance on external devices (eg, a separate workstation or server) and reduced end-to-end latency. A dedicated Unreal Engine (Epic Games, Cary, NC, United States) plugin was developed to interface with the packaged runtime, ensuring efficient model inference within the simulation environment.

### Model evaluation

The model was assessed for “clinical coherence”, which was operationalized as agreement with clinical data (RMSE, MAE, *R*^2^) and expert-rated plausibility of simulated physiology. For the latter part, 21 clinicians from the Leiden University Medical Centre participated in the model evaluation. Participants included perfusionists, intensivists, and residents with clinical involvement in ECMO management. All participants underwent VR ECMO training and completed a questionnaire regarding the perceived realism of the models during the simulation. The questionnaire was based on prior VR extracorporeal circulation simulation studies.^6^ The full questionnaire can be found in **Supplementary Material (S1)**. No patients were involved in the validation of the model.

This study is reported in accordance with the TRIPOD+AI statement, the checklist for which can be found in **Supplementary Materials (S8)**.^21^

## RESULTS

### Dataset characteristics

A total of 335 patients were included in the analysis, with a combined total of over 250 million data points collected. The dataset comprised 335 patients with ECMO data along with EHR recordings including laboratory values and medication administration events, used for Model 1. A subset of 113 patients had high-frequency vital monitor recordings, used for Model 2.

### Model performance

#### Overview.

The performance of the different models is summarized in **Tables 4 and 5**. The final neural network model is performed for both datasets with and without cannula size data and can be found in **Table 4**. **Table 5** provides the metrices of the second model. Furthermore, plots of both Models 1 and 2 are visualized in **Figure 2**, respectively.

**Figure 2.**
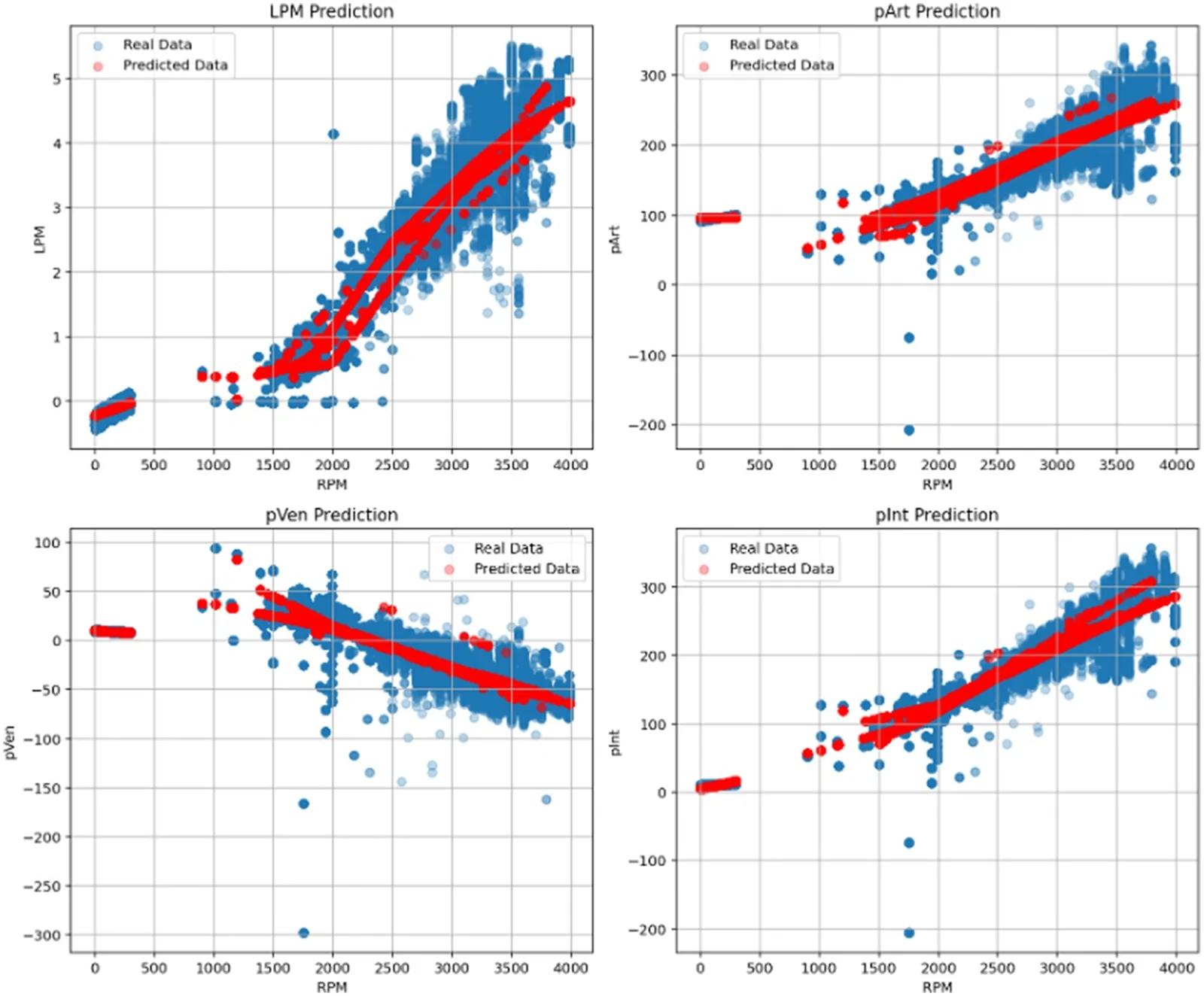
Predicted Versus Observed ECMO Circuit Outputs Generated by Model 1. Scatter plots display the relationship between pump revolutions per minute (RPM, *x*-axis) and 4 key circuit parameters (*y*-axis): blood flow in litres per minute (LPM, top left), arterial line pressure (pArt, top right), venous line pressure (pVen, bottom left), and inlet pressure (pInt, bottom right). Real data from the test set are shown in blue, while model-predicted values are overlaid in red

**Table 4.** Metrics for the Performance of Model 1, Predicting ECMO Machine-Related Variables, Demonstrating the Impact of Cannula Size on the Accuracy of the Predictions

Model 1 performance		Without cannula size			With cannula size		
Variable	Unit	RMSE	MAE	*R* ^2^	RMSE	MAE	*R* ^2^
Flow (LPM)	Litres per minute	0.53	0.38	0.18	0.32	0.26	0.57
Arterial pressure (pArt)	mm Hg	23.27	17.97	0.54	17.41	12.98	0.43
Venous pressure (pVen)	mm Hg	15.70	10.40	0.06	10.39	8.07	0.34
Internal pressure (pInt)	mm Hg	21.60	16.62	0.62	16.80	12.40	0.58

**Table 5.** Metrics for the Performance of Model 2, Predicting the Patient Vitals Based on ECMO Output Variables from Model 1

Output	Unit	RMSE	MAE
**Model 2 performance**			
Blood pressure	mm Hg	15.23	11.42
Heart rate	Beats per minute	19.50	15.14
Respiratory rate	Breaths per minute	5.78	4.40
Oxygen saturation	Percent	2.94	1.89
End tidal CO2	mm Hg	1.42	1.05

#### Cross-validation.

Five-fold cross-validation was performed to assess model robustness. Performance across folds was within an acceptable range, and results on the validation sets were comparable to those on the final test set. Neural network performance also was compared against linear regression baselines trained and evaluated using identical patient-level splits. Across cross-validation, neural networks demonstrated lower RMSE and MAE for flow, arterial pressure, and venous pressure, with comparable performance for inlet pressure. These results indicate that non-linear models provide added value over linear baselines for all ECMO circuit variables (see **Table S5**).

#### Scenario-specific performance.

For clinical disaster scenarios such as pump failure, the initial model required adjustment. Model behaviour in these cases was validated through the identification of clinical scenarios within the data that mirrored the test scenarios (cessation of ECMO support for example) and combined with empirical evaluation and expert assessment. Through targeted synthetic data augmentation at low RPMs, scenario outputs were improved to the point that expert reviewers judged them to be clinically plausible.

### VR model deployment and testing

All exported ONNX models performed inference with real-time latency in the VR simulation environment, consistently below the threshold required for real-time application.

Twenty-one clinicians involved in ECMO care participated in the VR evaluation, including 6 intensivists, 7 residents or fellows, and 8 perfusionists. The cohort was experienced, with 100% having ECMO experience and 59.1% independently managing ECMO, while 45.5% reported more than 10 years of clinical experience.

Feedback from the initial user testing sessions indicated that the simulated ECMO parameters were largely perceived as clinically coherent. A total of 86% of participants agreed that ECMO flow responded realistically to changes in pump speed, and 76% considered the displayed line pressures plausible. Monitor parameters were also judged positively, with 81% rating heart rate and oxygen saturation as plausible, 76% for arterial blood pressure, and 71% for end-tidal CO_2_. Integration of circuit and monitor data was considered helpful for decision-making by 81% of participants, and 76% reported that changes in ECMO settings produced the expected physiological responses. Further validation results can be found in **Supplementary Materials (S1)**. For the purposes of training, users felt that the model was sufficiently clinically accurate, with suggested refinements focused on more dynamic modelling of pulse pressure across different flow ranges and improving predictions at low RPM, where limited data may have contributed to inaccuracies.

## DISCUSSION

We developed and validated an end-to-end AI-driven methodology to (1) acquire and link high-frequency ECMO device and patient data, (2) construct a hierarchical, 2-model pipeline that models machine outputs and patient responses, and (3) deploy the resulting models for real-time inference in a VR training environment. The predictive errors for key variables were small and were judged to be clinically non-significant by the expert panel. This assessment held even in physiologically extreme scenarios, such as simulated ECMO pump failure in a fully VA-ECMO dependent patient. Quantitative comparison with existing rule-based or lumped-parameter ECMO models was not undertaken, as these models are predominantly evaluated using benchtop or experimental setups rather than clinical patient datasets, precluding direct performance benchmarking.

### Future outlook

Future work will prospectively expand the pipeline across centres, ECMO types and models, and ECMO modes while expanding inputs to continuous waveforms, ventilator and pump streams, blood gases, and medication doses ensuring sub-second level time-sync. Modelling will include hybrid, physics-based, and lightweight sequence architectures that preserve plausibility at physiological extremes, whilst representing an acceptable computational burden for mobile devices such as VR goggles. The absence of direct cardiac function measures will be addressed by approaches including live 12-lead electrocardiogram waveforms and deriving real-time left ventricular ejection fraction estimates using methodologies such as that of Devkota et al,^22^ enabling the simulation of patients with varying amounts of required circulatory support. Additional coverage of rare failure states will be performed through structural data collection a system of marking clinically significant events and/or retrospective identification failure events by clinical staff and researchers. Educational impact of the simulation will be tested in randomized controlled studies. To improve generalizability while protecting privacy, we will pilot federated learning across international centres, adopt interoperability standards, and release versioned pipelines with model cards. We will focus additionally on equity and bias mitigation within the database. Finally, the modular architecture will be extended to other mechanical circulatory support such as our validated VR ECC simulator.^6^

### Limitations

Our approach has several limitations. The model currently does not include a formal measure of ventricular ejection fractions due to the sparse and time unsynchronized nature of the readings, using only pulse pressure as a proxy for cardiac function. Similarly, the VV ECMO model does not include lung function for similar reasons. Our results reveal that the addition of clinical variables may improve the performance of some outputs, at the expense of others (such as in the case of adding cannula sizes), likely due to multicollinearity which exists between the variables. This is compounded by the fact that ECMO data are dominated by narrow operating ranges, with sparse coverage at clinically extreme values. Second, despite high-frequency capture, residual misalignment is possible, considering actions that may be registered retrospectively, such as the administration of a bolus of medication. Thirdly, the data from 2 centres have been included in the training set to create this model, limiting external validity. To strengthen external validity, we foresee federated learning across multiple centres, countries, and ECMO device vendors. This study was not registered in advance due to the iterative nature of development and the rapid changes of the VR technology.

Virtual reality head-mounted displays remain computationally constrained, with performance characteristics close to mobile devices. This imposes strict limits on neural network complexity. As a result, architectures must be highly optimized for efficiency, sometimes at the expense of physiological detail and therefore reducing the *R*^2^ of the models, to ensure smooth real-time simulation.

## CONCLUSION

We present an end-to-end, scalable methodology that combines high-resolution ECMO device data with patient clinical data to create a modular 2-stage predictive AI model that predicts the mechano-physiology of patients that undergo ECMO support, for training purposes. Trained with patient-level splits and cross-validation, the models produced clinically coherent, stable predictions across routine and extreme clinical disaster scenarios. The models were implemented in a VR environment in real time, enabling responsive, high-fidelity training without dedicated hardware. This data-driven “digital twin” complements mechanistic approaches, broadens access to ECMO education, and establishes a reproducible path from raw ICU data to deployable simulation. While external validity and rare-event coverage require further work, the framework provides a strong foundation for multicentre validation and data inclusion through federated learning, ultimately raising ECMO training fidelity and access internationally.

## Supplementary Material

ivag047_Supplementary_Data

## Data Availability

The data underlying this article cannot be shared publicly due to the national and local privacy requirements of the institutions in which the data was gathered, and the terms under which institutional review board ethical approval was granted. The metadata and research protocol will be shared on reasonable request to the corresponding author.
