# Supplementary material for: Building an Extracorporeal Membrane Oxygenation Digital Twin Using High-Resolution Patient Data: An Artificial Intelligence Model for Virtual Reality Simulation
Source: Interdiscip Cardiovasc Thorac Surg. 2026 Feb 19;41(11):ivag047. doi: 10.1093/icvts/ivag047 (PMC13746011; doi:10.1093/icvts/ivag047)
Supplement: Supplementary file 1 [file ivag047_supplementary_data.zip › S1.docx]

**S1. Physiology Questionnaire**

Please rate the following statements of a scale of 1 to 5, where 1 is fully disagree, 3 is neutral, and 5 is fully agree.

1. The ECG waveform appeared realistic.
2. The arterial blood pressure waveform appeared realistic.
3. The photoplethysmogram waveform appeared realistic.
4. The capnogram waveform appeared realistic.
5. I found the waveforms on the patient monitor helpful for decision-making.
6. The displayed heart rate was plausible.
7. The displayed arterial blood pressure was plausible.
8. The displayed peripheral oxygen saturation was plausible.
9. The displayed end-tidal CO2 was plausible.
10. The combination of waveforms and numerical values improved realism of the VR application.
11. The displayed ECMO flow responded realistically to changes in RPM.
12. The displayed ECMO line pressures (venous, pre-oxygenator, post-oxygenator) were plausible.
13. Observing ECMO parameters alongside patient monitor data helped me in decision-making.
14. Changes I made to ECMO settings affected the physiological values as expected.

**Figure S2:** Evaluation of the simulation of physiological data for the complete sample (*n* = 22). Results are presented for (A) integration with ECMO and decision support, (B) waveform realism, and (C) plausibility of numerical values. Abbreviations: ECG, electrocardiogram; ABP, arterial blood pressure; PPG, photoplethysmogram; HR, heart rate; SpO_2_, peripheral oxygen saturation; ETCO_2_, end-tidal carbon dioxide; RPM, revolutions per minute.

**S3: Data integration, preprocessing, and modelling pipeline**

1. Raw data acquisition

- ECMO device logs
  - Sampling interval: 5 minutes
  - Variables: RPM, flow, arterial pressure (pArt), venous pressure (pVen), inlet pressure (pInt)
- Electronic health record (EHR) data
  - Demographics, laboratory values, medication events
- High-frequency bedside monitor data (subset of patients)
  - Waveforms sampled at 25 Hz

2. Cohort definition

- Total patients identified: 335
- Patients with ECMO + EHR data: 335
- Patients with high-frequency bedside monitoring: 113
- Patients without high-frequency data excluded from Model 2
- No imputation of missing high-frequency vital signs

3. Timestamp alignment and synchronisation

- Manual matching of ECMO device data and EHR records using EHR timestamps and perfusion records
- Correction for device clock drift using:
  - Low-frequency ECMO variables recorded in the EHR
  - Reference events such as ECMO initiation and cessation
- Alignment validated by checking temporal concordance around reference events
- Records failing alignment excluded from downstream modelling (0 cases)

4. Temporal resampling

- ECMO device data interpolated to 30-second resolution
- Bedside monitor waveforms downsampled from 25 Hz to 30-second resolution
- All streams aligned to a common 30-second time grid

5. Missing data handling

- High-frequency vital signs
  - No imputation
  - Patients without these signals excluded from Model 2
- ECMO device variables
  - Interpolated only for resampling
- Cardiac function metrics (e.g. ejection fraction)
  - Excluded due to sparse and unsynchronised availability

6. Feature engineering

- Model 1 inputs:
  - RPM
  - Cannula sizes (arterial and venous)
- Model 1 outputs:
  - Flow
  - pArt, pVen, pInt
- Model 2 inputs:
  - Model 1 outputs
  - Selected patient vital signs
- Feature inclusion guided by domain expertise, literature, and iterative testing

7. Handling data imbalance and rare operating regimes

- Observed clustering around standard RPM ranges
- Sparse coverage at low RPM values
- Generation of sparse synthetic samples:
  - RPM range: 0–300
  - Physiologically plausible ranges for pressures and flow
  - Noise added
  - Distributed across pseudo-patients to avoid leakage
- Synthetic data used only to stabilise training at extremes

8. Train–validation–test splitting

- Patient-level splitting to prevent data leakage
- Approximate proportions:
  - Training (inside CV): ~64%
  - Validation (inside CV): ~16%
  - Test (held-out patients): 20%
- Test set:
  - 11 real patients + 1 synthetic patient
- Training set:
  - 68 real patients + 8 synthetic patients
- Stratification performed to preserve data-point balance rather than patient count

9. Model training

- Model 1:
  - Neural network (TensorFlow 2.19.0)
  - Optimizer: Adam
    - Learning rate: 0.001
  - Loss: mean squared error
  - Epochs: 5
  - Batch size: 256
  - Random seed: 5
- Baseline comparison:
  - Linear regression (scikit-learn 1.6.1)
- Model 2:
  - Standard linear regression
  - Random seed: 5

10. Model evaluation and validation

- Five-fold cross-validation
- Metrics computed:
  - MAE
  - RMSE
  - Standard deviation of error
  - R²
- Metric definitions:
  - Errors = y_true − y_pred
- Final evaluation on held-out patient test set
- Scenario-specific qualitative validation for disaster scenarios via expert review

**S4: Model Hyperparameters, Training Procedure, and Cross-Validation**

**Model hyperparameters**

Model 1 was implemented as a feedforward neural network using TensorFlow (version 2.19.0) with a fixed random seed of 5 to ensure reproducibility. Training was performed using the Adam optimiser with a learning rate of 0.001 and mean squared error as the loss function. Models were trained for five epochs with a batch size of 256. No explicit regularisation techniques, such as dropout or weight decay, were applied. All hyperparameters were fixed a priori and were not modified during cross-validation.

Model 2 was implemented as a standard linear regression model using scikit-learn (version 1.6.1), also with a fixed random seed of 5. Given the simplicity of the model, no additional optimisation parameters or regularisation methods were required.

**Training and validation strategy**

Model performance was assessed using five-fold cross-validation with patient-level splits to prevent information leakage. All data from a given patient were assigned exclusively to a single fold. Approximately 64% of patients were used for training, 16% for validation within cross-validation, and 20% were reserved as a held-out test set. Hyperparameter tuning was not nested within the cross-validation procedure, as no automated hyperparameter optimisation was performed. Cross-validation was used solely to estimate performance and robustness.

**Performance aggregation and metrics**

Model performance was quantified using mean absolute error (MAE), root mean square error (RMSE), standard deviation of the residuals (STD), and coefficient of determination (R²). Errors were defined as the difference between observed and predicted values. Average performance across cross-validation folds is reported together with the standard deviation of the residuals. Final model performance was evaluated separately on the held-out test set and is reported in the main manuscript.

**S5: Comparison of ECMO model versus linear regression models and variable variation in literature:**

| Output variable | Model | RMSE | MAE | STD | R² | Dataset SD | Literature  SD |
| --- | --- | --- | --- | --- | --- | --- | --- |
| Flow (LPM) | Neural network | 0.45 | 0.32 | 0.43 | 0.58 | 1,74 | 0.7-1.0 (1) |
| Flow (LPM) | Linear regression | 0.59 | 0.49 | 0.45 | 0.28 |  |  |
| Arterial pressure (pArt) | Neural network | 22.72 | 16.53 | 20.66 | 0.61 | 56,76 |  |
| Arterial pressure (pArt) | Linear regression | 30.53 | 23.63 | 26.47 | 0.29 |  |  |
| Venous pressure (pVen) | Neural network | 12.91 | 10.04 | 12.48 | 0.54 | 26,18 |  |
| Venous pressure (pVen) | Linear regression | 17.44 | 14.34 | 15.43 | 0.16 |  |  |
| Inlet pressure (pInt) | Neural network | 21.82 | 16.83 | 18.92 | 0.69 | 96,91 |  |
| Inlet pressure (pInt) | Linear regression | 23.62 | 17.87 | 21.14 | 0.64 |  |  |

1) Alwardt CM, Wilson DS, Alore ML, Lanza LA, Devaleria PA, Pajaro OE. Performance and Safety of an Integrated Portable Extracorporeal Life Support System for Adults. J Extra Corpor Technol. 2015 Mar;47(1):38-43. PMID: 26390678; PMCID: PMC4566819.

**Supplementary Table S6. Expert ratings summary and agreement**

Expert ratings were collected on a 5-point Likert scale (1 = Fully disagree, 5 = Fully agree). For each item, we report the mean, standard deviation (SD), and 95% confidence interval (CI). To quantify the level of expert consensus, we additionally report within-group agreement (*rwg*) per item (computed relative to a uniform null distribution for a 5-point scale).

**Supplementary Table S6A**

Subscale-level summaries (mean of items per respondent)

| **Scale** | **Items** | | **N** | | **Mean** | | **SD** | **95% CI** |
| --- | --- | --- | --- | --- | --- | --- | --- | --- |
| Waveform realism (ECG/ABP/PPG/Capnogram) | | 4 | | 22 | | 4.03 | 0.87 | 3.65–4.41 |
| Vital sign plausibility (HR/BP/SpO₂/etCO₂) | | 4 | | 22 | | 3.77 | 0.86 | 3.39–4.16 |
| ECMO response and plausibility | | 6 | | 22 | | 3.92 | 0.78 | 3.57–4.26 |
| Learning and confidence | | 4 | | 20 | | 3.52 | 0.65 | 3.22–3.83 |
| Satisfaction and recommendation | | 3 | | 22 | | 4.50 | 0.49 | 4.28–4.72 |
| Usability | | 5 | | 22 | | 4.13 | 0.52 | 3.90–4.36 |
| Immersion and engagement | | 4 | | 22 | | 4.33 | 0.49 | 4.11–4.55 |

| **Item** | **N** | **Mean** | **SD** | **95% CI** | **rwg** |
| --- | --- | --- | --- | --- | --- |
| The ECG waveform appeared realistic. | 21 | 4.19 | 0.81 | 3.82–4.56 | 0.67 |
| The arterial blood pressure waveform appeared realistic. | 22 | 4.00 | 1.02 | 3.55–4.45 | 0.48 |
| The photoplethysmogram waveform appeared realistic. | 18 | 4.17 | 0.86 | 3.74–4.59 | 0.63 |
| The capnogram waveform appeared realistic. | 16 | 4.12 | 0.62 | 3.80–4.45 | 0.81 |
| I found the waveforms on the patient monitor helpful for decision-making. | 21 | 3.90 | 0.94 | 3.48–4.33 | 0.55 |
| The displayed heart rate was plausible. | 22 | 3.77 | 0.97 | 3.34–4.20 | 0.53 |
| The displayed arterial blood pressure was plausible. | 22 | 3.59 | 0.91 | 3.19–3.99 | 0.58 |
| The displayed peripheral oxygen saturation was plausible. | 22 | 3.82 | 0.96 | 3.39–4.24 | 0.54 |
| The displayed end-tidal CO2 was plausible. | 22 | 3.91 | 0.81 | 3.55–4.27 | 0.67 |
| The combination of waveforms and numerical values improved realism of the VR application. | 22 | 4.23 | 0.87 | 3.84–4.61 | 0.62 |
| The displayed ECMO flow responded realistically to changes in RPM. | 22 | 3.86 | 0.83 | 3.49–4.23 | 0.66 |
| The displayed ECMO line pressures (venous, pre-oxygenator, post-oxygenator) were plausible. | 22 | 3.86 | 0.89 | 3.46–4.25 | 0.60 |
| Observing ECMO parameters alongside patient monitor data helped me in decision-making. | 22 | 4.05 | 0.90 | 3.65–4.45 | 0.60 |
| Changes I made to ECMO settings affected the physiological values as expected. | 22 | 3.91 | 0.75 | 3.58–4.24 | 0.72 |
| The monitor helped me understand the consequences of my ECMO management decisions. | 22 | 3.91 | 0.81 | 3.55–4.27 | 0.67 |
| I learned a lot from this simulation about how to deal with ECMO scenario. | 22 | 3.59 | 0.91 | 3.19–3.99 | 0.58 |
| The training helped me being more confident in managing ECMO patients. | 22 | 3.41 | 0.85 | 3.03–3.79 | 0.64 |
| The training helped me remember the steps in an ECMO scenario. | 22 | 3.68 | 0.89 | 3.29–4.08 | 0.60 |
| After the training, I have enough knowledge to take the lead in an ECMO scenario. | 20 | 3.43 | 0.75 | 3.08–3.78 | 0.72 |
| I enjoyed participating in this ECMO training. | 22 | 4.68 | 0.48 | 4.47–4.89 | 0.89 |
| I was satisfied with the quality of this training. | 22 | 4.36 | 0.79 | 4.01–4.71 | 0.69 |
| I would recommend this training to other colleagues. | 22 | 4.45 | 0.51 | 4.23–4.68 | 0.87 |
| The application is easy to use. | 22 | 4.32 | 0.65 | 4.03–4.60 | 0.79 |
| I learned to use it quickly. | 22 | 4.32 | 0.57 | 4.07–4.57 | 0.84 |
| The interaction with the software felt intuitive. | 22 | 4.05 | 0.72 | 3.73–4.37 | 0.74 |
| I can use the software without written instructions. | 22 | 3.95 | 0.95 | 3.53–4.37 | 0.55 |
| I was not distracted during the training. | 22 | 4.05 | 0.84 | 3.68–4.43 | 0.65 |
| The simulator was realistic. | 22 | 4.27 | 0.55 | 4.03–4.52 | 0.85 |
| I felt like I was in a real ICU. | 22 | 4.27 | 0.70 | 3.96–4.58 | 0.75 |
| I was interested in the progress of the events within the simulation. | 22 | 4.36 | 0.66 | 4.07–4.66 | 0.78 |
| I felt actively involved in the patient scenario of the training. | 22 | 4.41 | 0.50 | 4.19–4.63 | 0.87 |
